# Complete Genome Sequence and Methylome of *Salmonella enterica* subsp. *enterica* Cerro, a Frequent Dairy Cow Serovar

**DOI:** 10.1128/genomeA.01350-15

**Published:** 2016-01-28

**Authors:** Bradd J. Haley, Cary Pirone, Tim Muruvanda, Eric Brown, Marc Allard, Jeffrey S. Karns, Jo Ann S. Van Kessel

**Affiliations:** a Beltsville Agricultural Research Center, Environmental Microbial and Food Safety Laboratory, USDA-ARS, Beltsville, Maryland, USA; b Division of Microbiology, Office of Regulatory Science, Center for Food Safety and Nutrition, U.S. Food and Drug Administration, College Park, Maryland, USA

## Abstract

*Salmonella enterica* subsp. *enterica* serovar Cerro is an infrequent pathogen of humans and other mammals but is frequently isolated from the hindgut of asymptomatic cattle in the United States. To further understand the genomic determinants of *S*. Cerro specificity for the bovine hindgut, the genome of isolate CFSAN001588 was fully sequenced and deposited in the GenBank database.

## GENOME ANNOUNCEMENT

*Salmonella enterica* subsp. *enterica* is a frequent cause of morbidity and mortality in humans and animals. Although all strains of this subspecies are considered potential pathogens of humans, and many are pathogens of other animals, some serovars have consistently been isolated from their hosts in the absence of clinical signs of an infection (1, 2). *Salmonella enterica* subsp. *enterica* serovar Cerro is frequently isolated from asymptomatic dairy cows in the United States (3, 4). This serovar has also been shown to persist among members of a dairy herd for several years (5). The frequent isolation of this organism in the absence of observable signs of infections and its persistence in dairy herds suggest that strains of this serovar are adapted as commensal members of the bovine intestine. However, the mechanisms influencing specificity for the bovine intestine are not yet elucidated.

To further evaluate the genomic features that may play a role in the apparently reduced virulence of *S*. Cerro in cows and humans, as well as its adaptation to the bovine hindgut, the genome of *Salmonella enterica* subsp. *enterica* serovar Cerro strain CFSAN001588, isolated from the feces of a dairy cow in south-central Pennsylvania on 13 September 2004, was fully sequenced. The isolate was grown overnight in Luria-Bertani broth, and DNA was extracted using a Qiagen DNEasy kit (Qiagen Ltd., Crawley, United Kingdom). The genome was sequenced using the Pacific Biosciences (PacBio) RS II sequencing platform (Pacific Biosciences, Menlo Park, CA, USA). Briefly, a single 10-kb library was prepared following the Pacific Biosciences library preparation protocol and sequenced using the C2 chemistry on single-molecule real-time (SMRT) cells with a 90-min collection protocol. The data were assembled *de novo* using the HGAP software package and finished with Quiver (6). The methylation of adenine and cytosine residues was determined based on kinetic variations in nucleotide incorporation rates as previously described (7). This analysis was implemented via the RS_Modification_and_Motif_Analysis protocol of SMRT Analysis version 1.1. The assembly resulted in three contigs of sizes 4,651,400 bp (chromosome), 53,952 bp (plasmid pCFSAN001588_001), and 62,884 bp (plasmid pCFSAN001588_002), with coverage estimated at 126×. Sequence data were annotated using the NCBI Prokaryotic Genome Annotation Pipeline.

Four methylated N^6^-methyladenine (m6A) motifs were observed across the genome and plasmids. These included ATGC^m6^AT, CAG^m6^AG, G^m6^ATC, and VG ^m6^AACK (V = not T, K = G or T). ATGC^m6^AT, CAG^m6^AG, and G^m6^ATC have been observed previously in *Salmonella* and other bacterial genera (8). The VG ^m6^AACK motif should be investigated further to determine whether the ambiguous assignments of V and K are correct or artifacts of the analysis.

### Nucleotide sequence accession numbers.

This whole-genome sequence project has been deposited in DDBJ/ENA/GenBank under the accession numbers CP012833, CP012834, and CP012835. The versions described in this paper are the first versions, CP012833.1, CP012834.1, and CP012835.1.
